# The DIAMOND Model: Deep Recurrent Neural Networks for Self-Organizing Robot Control

**DOI:** 10.3389/fnbot.2020.00062

**Published:** 2020-09-15

**Authors:** Simón C. Smith, Richard Dharmadi, Calum Imrie, Bailu Si, J. Michael Herrmann

**Affiliations:** ^1^Institute of Perception, Action and Behaviour (IPAB), School of Informatics, University of Edinburgh, Edinburgh, United Kingdom; ^2^State Key Laboratory of Robotics, Shenyang Institute of Automation, Institutes for Robotics and Intelligent Manufacturing, Chinese Academy of Sciences, Shenyang, China; ^3^School of Systems Science, Beijing Normal University, Beijing, China

**Keywords:** deep neural networks, autonomous learning, homeokinesis, self-organizing control, robot control

## Abstract

The proposed architecture applies the principle of predictive coding and deep learning in a brain-inspired approach to robotic sensorimotor control. It is composed of many layers each of which is a recurrent network. The component networks can be spontaneously active due to the homeokinetic learning rule, a principle that has been studied previously for the purpose of self-organized generation of behavior. We present robotic simulations that illustrate the function of the network and show evidence that deeper networks enable more complex exploratory behavior.

## 1. Introduction

Deep neural architectures (Fukushima and Miyake, 1980; Hinton et al., 2006) have reached a level comparable to human performance in certain pattern recognition tasks (Krizhevsky et al., 2012). Also in robotic applications, deep networks gain more and more importance, from state abstraction to seamless end-to-end control in complex repetitive tasks (Levine et al., 2016). Moreover, it has been speculated whether deep feed-forward networks can account for some aspects of information processing in the mammalian visual system (Serre et al., 2007), which is not to say that the brain *is* nothing but a collection of deep neural networks. Quite to the contrary, the brain is known to have dynamical properties that are much richer than standard deep architectures:

- Biological neural systems consist of patches of interconnected neurons which also receive re-entrant connectivity via other patches.
- Spontaneous behavior can occur at any level of depth and may spread in either direction.
- Sensory inputs are not only providing information for decision about actions, but are also analyzed for effects of previous actions.
- A hierarchical organization enables lateral transferability and flexible compositionality.
- There is little use for supervised learning.

Based on these considerations, we propose here an architecture that combines the undeniable strengths of deep neural networks with *homeokinesis* (Der, 2001), an approach to meet requirements of autonomous robots (see section 2). Our work connects to (Carvalho and Nolfi, 2016) where the introduction of flexibility and plasticity in a neural controller showed a good effect in a cleaning task, however, mainly based on an evolutionary approach, whereas we aim at a more principled architecture that achieves an increased flexibility by a hierarchy of identical controllers. The autonomously generate activity of higher-lever controllers provide an intrinsic motivation (Oudeyer et al., 2007) for the lower ones. In this way, we are able to propose a more brain-like architecture which implicitly realizes a predictive coding principle, compare (Adams et al., 2013) for a related approach, at least in some parameter range, as discussed below. An early interesting comparison is provided by (Rusu et al., 2003) which presents a neuro-fuzzy controller for determining the behavior of a robot in a navigation task. Their architecture had a similarly layered structure, although the behaviors had to be predefined at a time when homeokinesis (Der, 2001) was just being developed. More recently, differential Hebbian learning was used to explore possible behaviors of a robot (Pinneri and Martius, 2018), presenting a more brain-like approach at the low level, whereas we aim a model that captures characteristics of the area-level organization of the brain.

In the following, we will consider first the homeokinetically controlled sensorimotor loop (Der, 2001) as the basic element of the proposed system (section 2). In this way, we incorporate a source of spontaneous activity. The composition of these elements in the DIAMOND (Deep Integrated Architecture for sensoriMotor self-Organization aNd Deliberation) architecture (section 3) will thus be able to generate activity at all levels and work in a fully self-supervised way, although it is also possible to steer the system to desired behavior by very small guiding inputs (Martius and Herrmann, 2011). The main layout of the architecture includes a basic layer that receives information from outside world and sends actions and is expected to represent low-level features. There is a variable number of deeper layers that interact only with the neighboring layers and which represent more abstract features that are extracted from the data through the lower layers. The architecture learns by the homeokinetic learning rule (see below) which implies that consistency between neighboring layers is required. We will present a few experimental results in section 4, and discuss the realism and performance of the architecture as well as further work in section 5.

## 2. Homeokinetic Control

The basic element of our architecture is formed by a homeokinetic controller, which we will describe here only briefly, details can be found in (Der and Martius, 2012). This unsupervised active learning control algorithm shapes the interaction between a robot and its environment by updating the parameters of a controller and of an internal model. The learning goal can be characterized as a balance of predictability and sensitivity with respect to future inputs. The resulting behavior is random yet coherent both temporally and across multiple degrees of freedom. The controller is a parametric function

(1) $$\begin{array}{l} {\text{y}}_{t}=C\left({\text{x}}_{t};\text{C}\right) \end{array}$$

of the vector **x**_*t*_ of current sensory states of the robot. It generates a vector of motor commands **y**_*t*_ in dependence on the current values of the parameter matrix **C**. The update of the parameters is based on the sensitivity of the distance between inputs and their predictions by means of an internal model. This model

(2) $$\begin{array}{l} {\hat{\text{x}}}_{t+1}=M\left({\text{x}}_{t},{\text{y}}_{t};\text{M}\right), \end{array}$$

produces a prediction of future states ${\hat{\text{x}}}_{t+1}$ based on the current input **x**_*t*_ or action **y**_*t*_ or both, and a parameter matrix **M**. The difference between actual and estimated state defines the prediction error

(3) $$\begin{array}{l} \xi_{t+1}={\text{x}}_{t+1}-{\hat{\text{x}}}_{t+1}, \end{array}$$

which gives rise to one of the two complementary objective functions that are relevant here, firstly the prediction error

(4) $$\begin{array}{l} E_{t+1}=||{\text{x}}_{t+1}-{\hat{\text{x}}}_{t+1}{||}^{2}, \end{array}$$

which is used to adapt the parameters **M** of the internal model (2), and secondly the *time loop error*

(5) $$\begin{array}{l} {\text{E}}_{t}=||{\text{x}}_{t}-{\hat{\text{x}}}_{t}{||}^{2}, \end{array}$$

which is based on a post-diction ${\hat{\text{x}}}_{t}$ of previous input **x**_*t*_ obtained via the inverse of Equation (2) given the new input **x**_*t*+1_, i.e., **E**_*t*_ is calculated only at time step *t*+1, and is related to the prediction error (4) by

(6) $$\begin{array}{l} {\text{E}}_{t}=||\eta_{t}{||}^{2}=\eta_{t}^{⊤}\eta_{t}=\xi_{t+1}^{⊤}{\left(J_{t}{J_{t}}^{⊤}\right)}^{-1}\xi_{t+1}. \end{array}$$

where *J* is the linearization of the maps from current input to next input dependent on the current controller. As only the projection **η** of *J*^−1^ on **ξ** is relevant, the time loop error can be efficiently estimated. The homeokinetic learning rule updates the parameter matrix **C** of the controller (1) by gradient descent

(7) $$\begin{array}{l} \Delta C_{ij}=-\varepsilon_{\text{C}}\frac{\partial {\text{E}}_{t}}{\partial C_{ij}}, \end{array}$$

where *C*_*ij*_ is an element of **C** and ε_**C**_ is a learning rate.

If the representational power is of less importance than the flexibility (Smith and Herrmann, 2019), then a simple quasi-linear system can be considered as sufficient. Below, when we will consider a multi-layered system, the representational power is meant to be achieved by the interaction between the layers each of which will consist of one instance of the current controller-predictor unit. A pseudo-linear controller, i.e., a quasi-linear function of the inputs with coefficients that are adaptive on the behavioral time scale,

(8) $$\begin{array}{l} y_{t}=C\left({\text{x}}_{t}\right)=g\left(\text{C}{\text{x}}_{t}+\text{c}\right) \end{array}$$

and a linear model

(9) $$\begin{array}{l} {\hat{\text{x}}}_{t+1}=M\left({\text{y}}_{t}\right)=\text{M}{\text{y}}_{t}+\text{m}, \end{array}$$

does thus not limit the complexity of achievable control. The parameters of the controller and the model are now the matrices **C** and **M** resp., which are complemented by the matching bias vectors **c** and **m**. In order to incorporate limitations of actions of the robot, the controller is quasi-linear due to the element-wise sigmoidal function *g*. Because of the simple structure of Equation (8), we can omit here the state dependency (2) and define the model *M* only in motor space. The parameter update (7) becomes

(10) $$\begin{array}{l} \Delta C_{ij}=\varepsilon_{\text{C}}\eta^{⊤}J\frac{\partial J}{\partial C_{ij}}\eta , \end{array}$$

and analogously for the bias term **c**. With **μ** = **G**′**M**^⊤^(*J*^⊤^)^−1^**η** and **ζ** = **Cη** the learning rules for a linear controller with a linear model are

(11) $$\begin{array}{l} \Delta C_{ij}=\varepsilon_{\text{C}}\mu_{i}\eta_{j}-2\varepsilon_{\text{C}}\mu_{i}\zeta_{i}y_{i}x_{j} \end{array}$$

(12) $$\begin{array}{l} \Delta c_{i}=-2\varepsilon_{\text{C}}\mu_{i}\zeta_{i}y_{i}. \end{array}$$

Simultaneously, but possibly with a different learning rate, the parameters **M** of the linear model (9) are updated via gradient descent on the standard prediction error (Equation 4, rather than Equation 6).

(13) $$\begin{array}{l} \Delta M_{ij}=-\varepsilon_{\text{M}}\frac{\partial E}{\partial M_{ij}}=\varepsilon_{\text{M}}\xi_{i}y_{j} \end{array}$$

(14) $$\begin{array}{l} \Delta m_{i}=-\varepsilon_{\text{M}}\frac{\partial E}{\partial b_{j}}=\varepsilon_{\text{M}}\xi_{i} \end{array}$$

where ε_**M**_ is the learning rate for the adaptation of the internal model. The ratio of the two learning rates ε_**C**_ and ε_**M**_ is known to be critical for the behavior of controlled robot (Smith and Herrmann, 2019). For the architecture presented next, an optimized ratio is to be used, see also Figure 2.

## 3. The DIAMOND Model

### 3.1. Deep Homeokinesis

**Figure 1:**
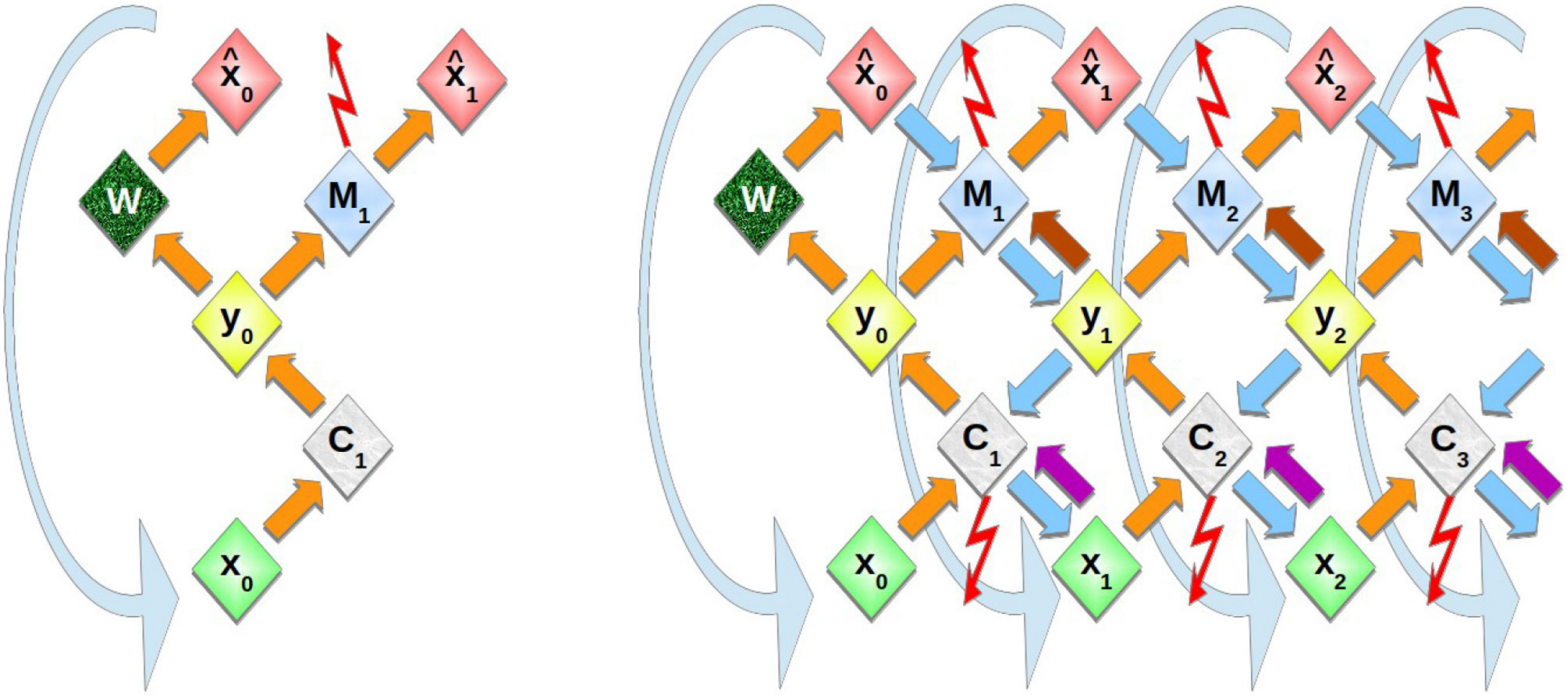
Schematic representation of multi-layer homeokinetic learning. **Left**: In the elementary sensorimotor loop, a control action **y**_0_ is calculated by the controller *C*_1_ and executed in the environment *W* which then produces the new input ${\hat{\text{x}}}_{0}$. The prediction error is obtained as the difference of new sensory input ${\hat{\text{x}}}_{0}$ and its prediction ${\hat{\text{x}}}_{1}$ that was obtained from the previous input ${\hat{\text{x}}}_{0}$. It is used in the update of the model *M*, see Equations (13), (14). **Right**: In homeokinetic learning, the time-loop error, i.e., the difference of previous input **x**_0_ and re-estimated previous input **x**_1_ (which is obtained via the downwards arrows and corresponds to ${\hat{\text{x}}}_{t}$ in Equation 5), is used to update the controller parameters, see Equations (11), (12). The curved downward arrows indicate the time step: The “new” input that was previously predicted or obtained from the environment, is now used by the controller to produce the next action (rather than the re-estimated input). The inner layers follow exactly the same dynamics based on predictions from the respective outer layers rather than based on the environment. Top-down effects are included by additional connections This includes virtual actions (arrows from **y**_*i*_ to *M*_*i*_) analogous to the initiation of actions in the environment, and virtual states taken into account by the controller (arrows from **x**_*i*_ to *C*_*i*_). The activities are propagated alternatingly through the upwards (orange, violet, and brown) arrows and through the respective transposed matrices via downwards arrows (cyan), both of which correspond to a set of parallel fibers, whereas the adaptive interconnections are maintained in the controller (**C** nodes) or the model (**M** nodes).

The DIAMOND model is a generalization of the homeokinetic controller described in section 2. As shown in Figure 1, the comparison of a state variable **x**(*t*) and its estimate $\hat{\text{x}}\left(t\right)$ is now repeated also for estimates of estimates etc., **x**_0_(*t*) = **x**(*t*), ${\text{x}}_{1}\left(t\right)=\hat{\text{x}}\left(t\right)$, **x**_2_(*t*), … , where each pair of neighboring layers corresponds to a homeokinetic controller that acts onto the lower layer as its environment and receives biases from the higher layer. In the inner layers (larger ℓ) the external information becomes less and less dominant.

In order to use homeokinetic learning in a multilayer architecture, several instances of the homeokinetic sensorimotor loop are stacked. The internal model of any lower layer serves as the “world” for the next higher layer. Likewise, estimates for input obtained at by a lower layer are the inputs for the higher layers, so each layer reproduces the elementary loop shown in Figure 1.

### 3.2. Simple Variant

The architecture consists of controllers for each layer ℓ < *L* (no controller for ℓ = *L*)

(15) $$\begin{array}{l} {\text{y}}_{\ell }\left(t\right)=C_{\ell +1}\left({\text{x}}_{\ell }\left(t\right)\right)=g\left({\text{C}}_{\ell +1}{\text{x}}_{\ell }\left(t\right)+{\text{c}}_{\ell +1}\right) \end{array}$$

and linear models that are given by

(16) $$\begin{array}{l} {\hat{\text{x}}}_{\ell }\left(t+1\right)=M_{\ell }\left({\text{y}}_{\ell -1}\left(t\right),{\text{y}}_{\ell }\left(t\right)\right)\\ \;={\text{M}}_{\ell }{\text{y}}_{\ell -1}\left(t\right)+{\tilde{\text{M}}}_{\ell }{\tilde{\text{y}}}_{\ell }\left(t\right)+{\text{m}}_{\ell }. \end{array}$$

which simplifies for the top layer ℓ = *L* where ${\tilde{\text{y}}}_{L}\left(t\right)\equiv 0$, i.e., no higher effects are present.

In Equation (16) also the effect of virtual actions ${\tilde{\text{y}}}_{\ell }\left(t\right)$, ℓ ≥ 1 is included as follows: First, the previous prediction of a layer ${\hat{\text{x}}}_{\ell }\left(t-1\right)$ is copied into the input unit **x**_ℓ_(*t*) at the beginning of the new time step, see Figure 1. The back-propagated input ${\hat{\text{x}}}_{\ell }\left(t-1\right)$ that was used in Equations (5) and (6) is no longer needed. From **x**_ℓ_(*t*) a virtual action **y**_ℓ_(*t*) is computed that then contributes additively to the prediction (16). The controller update is here the same as for the one-layer model, and the $\tilde{\text{M}}$ matrix (not shown in the figures) is updated in the same way as the **M** matrix.

### 3.3. Main Variant

The variant with extra connections (Figure 1) has for the controller

(17) $$\begin{array}{l} y_{\ell }\left(t\right)=C_{\ell +1}\left({\text{x}}_{\ell }\left(t\right)\right)\\ \;=g\left({\text{C}}_{\ell +1}{\text{x}}_{\ell }\left(t\right)+{\tilde{\text{C}}}_{\ell +1}{\hat{\text{x}}}_{\ell +1}\left(t-1\right)+{\text{c}}_{\ell +1}\right) \end{array}$$

i.e., in the same way as new input ${\hat{\text{x}}}_{0}\left(t+1\right)$ that is used to calculate the prediction error is also used in the next time step as input **x**_0_(*t*), we are also for ℓ > 0 using previous predictions as new virtual input. For the deepest layer ℓ = *L*, Equation (17) is not applied, and for the penultimate layer we have simply

(18) $$\begin{array}{l} y_{\ell }\left(t\right)=C_{\ell +1}\left({\text{x}}_{\ell }\left(t\right)\right)=g\left({\text{C}}_{\ell +1}{\text{x}}_{\ell }\left(t\right)+{\text{c}}_{\ell +1}\right). \end{array}$$

For the model, Equation (16) is used as above.

While the first **C** matrix in Equation (17) is adapted learned in the standard way (see Equations 11 and 12), the matrix $\tilde{\text{C}}$ is updated by gradient descent with respect to the prediction error for the action

$$E={\left({\text{y}}_{\ell }\left(t\right)-{\tilde{\text{y}}}_{\ell }\left(t\right)\right)}^{2},$$

where

$${\tilde{\text{y}}}_{\ell }\left(t\right)=g\left({\tilde{\text{C}}}_{\ell +1}{\hat{\text{x}}}_{\ell +1}\left(t-1\right)+{\tilde{\text{c}}}_{\ell +1}\right),$$

i.e., the input ${\hat{\text{x}}}_{\ell +1}\left(t-1\right)$ from the more inner level is used to predict the motor output **y**_ℓ_(*t*). The update equations for $\tilde{\text{C}}$ are similar to Equations (13) and (14), but also contains a derivative of *g*. Note that no loops are present in the network of Figure 1, which may not be a problem as the loops have no function (yet), and may be included later. However, it is not clear what “deliberation” could mean without these loops.

We assume that the inner (deeper) layers are updated first. The deepest layer ℓ = *L* has no variables, just the controller and the model. According to Equation (18), no higher-level input variables are needed in order to update the variables at ℓ = *L* − 1. In this way, virtual actions and virtual inputs are available to be used in Equations (17) and (16) to update the next layer toward the outer side, i.e., with lower ℓ. For the update of the matrices **M**, $\tilde{\text{M}}$, **C** and $\tilde{\text{C}}$ the time order is not essential, if the variables are calculated as described above.

### 3.4. Main Variant With Deep Associations

As a further variant, which is, however, not implemented in the present simulations, a standard deep neural network can be employed to connecting the inputs *x*_ℓ_ directly between neighboring levels. In this case a separate set of connections **P**_ℓ_ would be learned for map from *x*_ℓ−1_ to *x*_ℓ_. The weights **P** are learned by the activations **x**_ℓ_ that arise due to the activations of the network. In addition it is possible to add a further set of connections **R** that play the same role as **P**, but for the predicted sensor values.

The network can sustain persistent activity that represents an action perception cycle. Activity in the subnetworks that are completed by recurrent connections arises by self-amplification of noise or spurious activity following the homeokinetic learning of the respective controller. It may be possible to use also the cycles more explicitly for learning, but we want to restrict ourselves here to one-step learning rule, i.e., gradients are calculated only over one The full model also includes perceptual pathways consisting of bridges between input-related units. In this way the network activity becomes shaped by standard deep feed-forward networks.

## 4. Experimental Results

### 4.1. Active Response by the Recurrent Network

**Figure 2:**
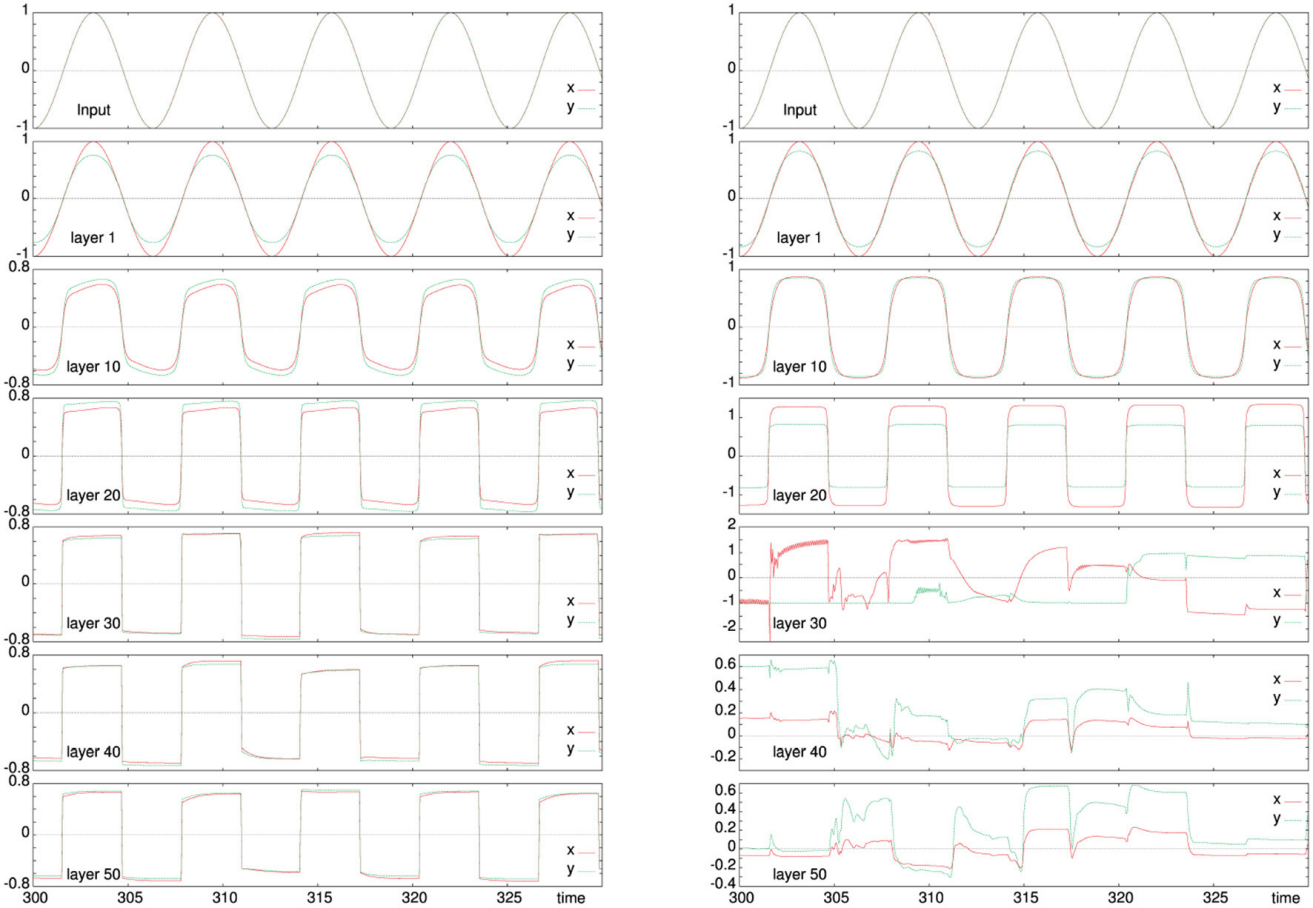
Activity evolution in a perceptually connected network structure according to the model in section 3.2. The sensory trajectory is shown by the solid line (red) and the intermediate motor action by the dashed line (green). The top row gives the input activity, the second row the activity of the first layer and the following rows show every 10th layer of the architecture to a total depth of 50. The left panel is for learning rates ε_**M**_ = 0.01, ε_**C**_ = 0.05, and the right one for ε_**M**_ = 0.1, ε_**C**_ = 0.2. While at low leaning rates, the input is similar across all layers, for larger ratios ε_**M**_/ε_**C**_ the model is more flexible and the deeper activity becomes largely independent on the input, which allows for self-organized activity in the deeper layers that is not immediately affecting the outside world.

As a first test, we have considered the simple variant of the architecture (see section 3.2) when it is driven with a sinusoidal input and the “world” reproduces simply a noisy version of the motor action as next input to the robot. Typical results are shown in Figure 2 for a two combinations of the learning rates ε_**C**_ (11, 12) and ε_**M**_ (13, 14), which lead either to an abstracted reproduction of the input in the deeper layers or to a self-organization of activity that, however remains without effect in this simple variant. At lower learning rates (left column), even deeper layers respond to the original input. In this case, the internal layers are square versions of the original input. For larger learning rates (right column), the internal layers have a different response. The fifth row shows a combination of homeokinetic adaptation (the red line between 310 and 320 s) and noisy output while still following the input from the first layer. Deeper layers (lower rows), tend have a decay in the generation of motor action attributed to the squashing function.

### 4.2. A Wheeled Robot in the Hills

**Figure 3:**
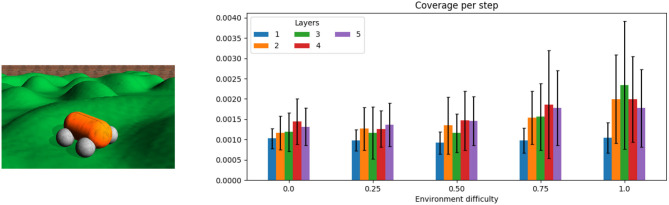
A four-wheeled robot exploring a hilly landscape. (See text and Smith and Herrmann, 2019 for details on the task). The panel on the right shows results for five levels of difficulty (linear scaling of the slopes, with the simples level being a flat ground) and five depths of the network (ℓ= 1, 2, 3, 4, 5) are considered, showing an increased exploration capability. The code for simulator (Der and Martius, 2012) and the DIAMOND controller architecture described here is available at https://github.com/artificialsimon/diamond.

The main variant (section 3.3) is used in an exploration task, where a four-wheeled robot is expected to cover a large portion of an unknown territory (Smith and Herrmann, 2019). The hilly landscape shown left in Figure 3 can be scaled in vertical direction such that different levels of difficulty can be achieved ranging from a flat ground (level 0) to slopes that require maximal motor power (level 1) and that can cause instabilities and thus large prediction errors (4). The activity decays in a five-layer DIAMOND model for a flat arena, as the inner layers are not needed, whereas for a hilly landscape (difficulty level > 0) the inner layers did not show much attenuation. The behavior of the robot is evaluated based on a 10 × 10 grid overlaid to the square-shaped arena. The number of visited grid cells is averaged over five runs for each difficulty and each controller depth and represented as a coverage rate. The total coverage was in all cases below 50% such that the increase of the coverage with time was nearly linear.

Whereas a single layer can achieve a similar performance across all terrain difficulties, for increasing difficulty of the task the higher layer are more and more engaged and take advantage of the increased errors in the terrain that provide thus a potential for a more comprehensive coverage of the arena per time unit.

### 4.3. A Spherical Robot in a Polygonal Arena

**Figure 4:**
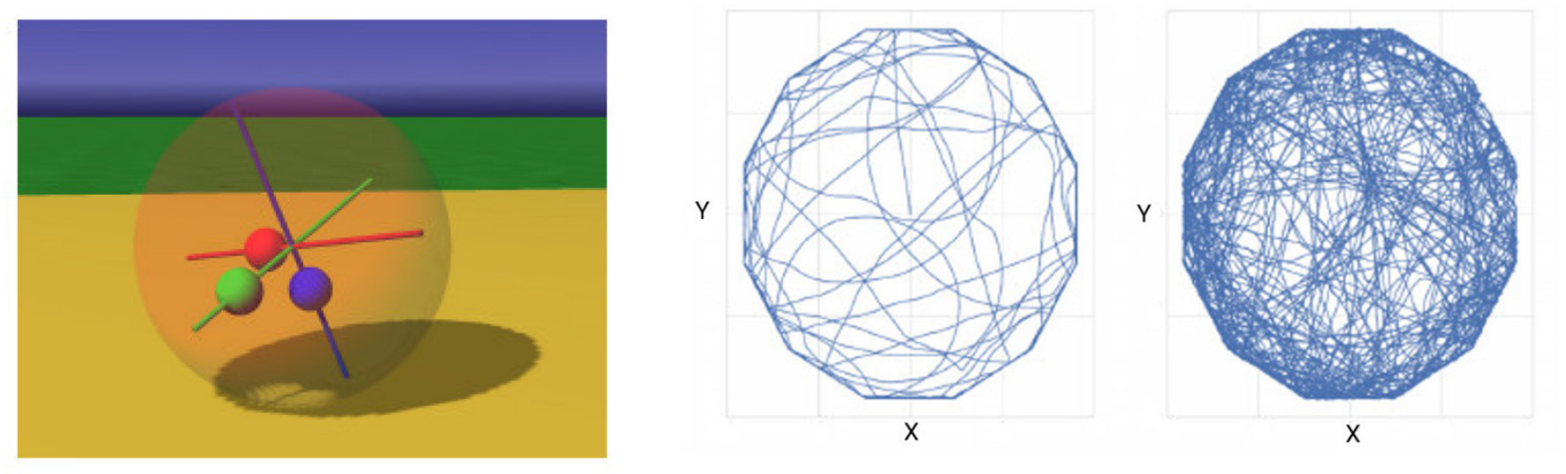
Spherical robot **(left)** in a dodecagonal arena. For a one-layer architecture, the robot mostly follows the wall **(middle)**, while for a 3-layer network, the robot shows a highly exploratory behavior **(right)**.

Finally, we studied a simulated spherical robot which is controlled by three masses that a movable along internal axes, see Figure 4, left. The robot is exploring freely in an polygonal environment which was chosen to discourage circular movement along the wall. The controller picks up quickly a suitable rhythm of the internal weights that is effecting in moving the robot in any direction. Collisions with wall usually stop the robot until the emergence of a different mode of the movements of the internal weights moves the robot in a different direction. Although a more systematic study is yet to be performed, it is already obvious that adding a small number of additional layers increases the behavioral repertoire of the robot and reduces the duration of any wall collisions and re-emergence of behavior in the robot. The example is also meant to demonstrate, that the applications of the learning rule and architecture are beyond exploration of a planar arena and can be used in order to generate and to organize elementary robotic behaviors.

## 5. Discussion

The numerical results seem to imply that a few layers are sufficient, i.e., a larger number of layers does not lead to further improvements or may require a much longer learning time than attempted here. It should, however, be considered that the tasks and environments are all very simple, such that it is not possible to generalize this observation to more complex situations. It can nevertheless be expected that the spontaneous internal activations that were observed for suitable learning rate ratios, lead to a learning time that is approximately linearly increasing with the number of layers, and not much worse. This is suggested by earlier results with homeokinetic learning rule (Martius et al., 2007).

The present model is a representation of the idea (see e.g., Anderson et al., 2012) that it is difficult to define a clear boundary between brain and body or even between body and world. At all layers the system follow the same principles in its adaptation of the actions onto lower layers and in the learning of a model that affects higher layers. The reduction of complexity of the internal dynamics toward higher layers is counterbalanced by the autonomous activity such that the main eigenvalue at each layer will hover near unity (Saxe et al., 2014).

Although the activity is updated here in parallel in all layers, the stacked structure is clearly similar to the subsumption architecture (Brooks, 1986) as it allows for shorter or longer processing loops. It remains to be studied whether more general architectures are beneficial, especially when more complex tasks are considered.

In Figure 1, it is understood that the dynamical variables (*x*, *y*, and $\hat{x}$) exist each in two instances, one updated by the controlling and predictive pathways, the other by the feedback within the re-estimation system. The need to disambiguate these units points to an interesting parallel to the roles of the layers of the mammalian cortex.

Finally, it should be remarked the principle of predictive coding is inherent in the architecture from the homeokinetic principle. Activity can only travel to the deeper layers if it is not already predicted by the internal model of the current layer. In some cases this can lead to a complete decay of the activity in the deeper layers (see Figure 3), although more complex robots and more challenging environments need to be studied in order to precisely identify parallels to the predictive coding principle in natural neural systems.

## Data Availability Statement

All datasets generated for this study are included in the article/supplementary material.

## Author Contributions

JH, BS, and SS: conception. JH and SS: model design. RD, SS, and CI: experiments. JH, BS, and SS: writing. JH: project organization.

## Conflict of Interest

The authors declare that the research was conducted in the absence of any commercial or financial relationships that could be construed as a potential conflict of interest.
